# Should high risk patients with concomitant severe aortic stenosis and mitral valve disease undergo double valve surgery in the TAVR era?

**DOI:** 10.1186/s13019-017-0688-z

**Published:** 2017-12-29

**Authors:** Pey-Jen Yu, Allan Mattia, Hugh A. Cassiere, Rick Esposito, Frank Manetta, Nina Kohn, Alan R. Hartman

**Affiliations:** 1 Department of Cardiovascular and Thoracic Surgery, Hofstra Northwell School of Medicine, 300 Community Drive, 1DSU, Manhasset, NY 11030 USA; 2 The Feinstein Institute for Medical Research, Manhasset, NY USA

**Keywords:** Aortic stenosis, Aortic valve surgery, Mitral valve surgery, Transcatheter aortic valve replacement

## Abstract

**Background:**

Significant mitral regurgitation in patients undergoing transcatheter aortic valve replacement (TAVR) is associated with increased mortality. The aim of this study is to determine if surgical correction of both aortic and mitral valves in high risk patients with concomitant valvular disease would offer patients better outcomes than TAVR alone.

**Methods:**

A retrospective analysis of 43 high-risk patients who underwent concomitant surgical aortic valve replacement and mitral valve surgery from 2008 to 2012 was performed. Immediate and long term survival were assessed.

**Results:**

There were 43 high-risk patients with severe aortic stenosis undergoing concomitant surgical aortic valve replacement and mitral valve surgery. The average age was 80 ± 6 years old. Nineteen (44%) patients had prior cardiac surgery, 15 (34.9%) patients had chronic obstructive lung disease, and 39 (91%) patients were in congestive heart failure. The mean Society of Thoracic Surgeons Predicted Risk of Mortality for isolated surgical aortic valve replacement for the cohort was 10.1% ± 6.4%. Five patients (11.6%) died during the index admission and/or within thirty days of surgery. Mortality rate was 25% at six months, 35% at 1 year and 45% at 2 years. There was no correlation between individual preoperative risk factors and mortality.

**Conclusions:**

High-risk patients with severe aortic stenosis and mitral valve disease undergoing concomitant surgical aortic valve replacement and mitral valve surgery may have similar long term survival as that described for such patients undergoing TAVR. Surgical correction of double valvular disease in this patient population may not confer mortality benefit compared to TAVR alone.

## Background

Aortic stenosis is the most prevalent valvular heart disease referred for treatment and it is frequently associated with concomitant mitral regurgitation (MR) [1]. Surgical aortic valve replacement is the standard treatment for symptomatic severe aortic stenosis, and there is general consensus that in the presence of severe MR, a double-valve operation is indicated. With the advent of the transcatheter aortic valve replacement (TAVR), surgical aortic valve replacement has largely been replaced by TAVR for patients at high or prohibitive surgical risk. It has been estimated that the prevalence of moderate or severe MR in patients undergoing TAVR ranges from 22 to 48% [1–6]. In these patients, the concomitant significant MR is typically left untreated and is associated with increased morbidity and mortality after TAVR [3, 4, 6–9].

Given the poor outcome of TAVR patients with severe aortic stenosis and significant MR, we sought to determine the short and long term outcomes of high risk patients undergoing concomitant surgical aortic valve replacement and mitral valve surgery to determine if open surgical approach may be preferable to TAVR only in such patients.

## Methods

This study was conducted with the approval of the Hofstra Northwell Health System Institutional Review Board with specific waiver of the need for individual patient consent. A retrospective review was performed on all patients who underwent concomitant aortic valve replacement and mitral valve surgery between 2008 and 2012. Each patient’s baseline surgical risk was determined using the Society of Thoracic Surgery Predicted Risk of Mortality (STS PROM) calculator. As the calculator is unable to give mortality risks for double valve surgery, the mortality risk for isolated AVR was used for each patient. Total predicted risk of mortality was calculated as the summation of the STS PROM plus any incremental risks not are not included in STS PROM [10, 11]. Patients were included in the study if their total predicted risk of mortality was greater than or equal to 8%. Patients undergoing aortic valve replacement for aortic insufficiency and/or aortic valve endocarditis were excluded. Definitions used for the preoperative risk factors and perioperative complications are standardized based on published guidelines by the New York State Department of Health for the New York State Cardiac Surgery Reporting System and the Society of Thoracic Surgeons cardiac surgery database. The following data were collected: gender, left ventricular ejection fraction, preoperative dialysis, presence of comorbidities (cerebral vascular disease, diabetes mellitus, hypertension, hyperlipidemia, congestive heart failure, chronic obstructive lung disease, peripheral vascular disease), urgency of surgery, previous myocardial infarction, preoperative arrhythmia and if this was a re-operation.

Clinical endpoint was long-term survival. Long-term survival was determined from the Social Security Death Index. All statistical analyses were performed using SAS Version 9.3 (SAS Institute Inc., Cary, NC). For categorical factors, survival from date of surgery was estimated using the Kaplan-Meier product limit method and compared using the log-rank test. For each continuous factor, Cox regression was used to determine if that factor was associated with survival. For this analysis, patients who died during surgery were counted as having survived for one day from the start of surgery. Length of stay (LOS) from day of surgery was calculated as the number of days from surgery to discharge, and patients who died during surgery were counted as having a one day LOS.

## Results

A total of 43 high-risk patients underwent aortic valve replacement for aortic stenosis and mitral valve surgery between 2008 and 2012. The preoperative characteristics for the study group are listed in Table 1. The average age was 80 ± 6 years old. Nineteen (44.2%) patients had prior cardiac surgery, 15 (34.9%) patients had severe chronic obstructive lung disease, 39 (90.7%) patients had congestive heart failure and 14 (32.6%) patients had severe pulmonary hypertension with pulmonary artery pressures ≥ 60 mmHg. The average STS PROM for isolated aortic valve replacement for the cohort was 10.1% ± 6.4%. The average total predicted risk of mortality which includes incremental risk factors not accounted for in the Society of Thoracic Surgeons risk model was 14.6% ± 6.9%.

**Table 1:** Pre-Operative Characteristics of the Patient Population

Pre-Operative Characteristics	Number (Percent) *n* = 43
Male	15 (34.88)
Body Mass Index	26.7 ± 7.1
Urgent Procedure	31 (72.09)
Re-Operation	19 (44.19)
Severe Chronic Obstructive Lung Disease	15 (34.88)
Creatinine	1.7 ± 1.2
Dialysis Dependent	5 (11.63)
Diabetes	17 (39.53)
Hypertension	39 (90.70)
Dyslipidemia	28 (65.12)
Cerebrovascular Disease	10 (23.26)
Peripheral Vascular Disease	7 (16.28)
Previous MI	7 (16.28)
Atrial Fibrillation/Flutter	17 (39.53)
Congestive Heart Failure	39 (90.70)
Age (years)	80 ± 6
Pulmonary Hypertension (≥60 mmHg)	14 (32.56)
Ejection Fraction	49.9 ± 13
STS PROM for isolated AVR	10.1 ± 6.4
Total Predicted Risk of Mortality for isolated AVR	14.6 ± 6.9

Postoperative morbidity is shown in Table 2. Overall operative mortality was 11.6%. Nineteen (44.2%) patients required prolonged ventilation, 10 (23.3%) patients had new renal failure with 6 (14.0%) patients requiring dialysis. Median time to postoperative extubation was 21.5 h (interquartile range 12 h – 141 h), median length of intensive care unit stay was 136.0 h (interquartile range 46 h − 586 h), and median length of hospital stay from day of surgery was 13 days (interquartile range 9 days - 24 days). Of the 38 patients who survived to discharge, 12 (31.6%) patients were discharged to home.

**Table 2:** Postoperative Complication Rates of the Patient Population

Postoperative Complications	Number (Percent)
Composite Morbidity	30 (69.77)
Operative Mortality	5 (11.63)
Re-Op Bleeding	1 (2.33)
Re-Op non Cardiac Cause	12 (27.91)
Deep Sternal Wound Infection	0 (0.00)
Sepsis	2 (4.65)
Stroke	1 (2.33)
Prolonged Ventilation	19 (44.19)
Pneumonia	1 (2.33)
Renal Failure	10 (23.26)
Dialysis Required	6 (13.95)
Cardiac Arrest	2 (4.65)

**Fig. 1:**
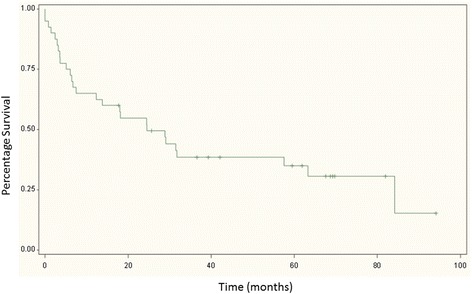
Kaplan Meier Log Rank Survival Curve

The mortality rate was 23% at six months, 33% at 1 year and 42% at 2 years (Fig. 1). There were no associations between preoperative risk factors and survival (Table 3).

**Table 3:** Association between Preoperative Risk Factors and Survival

Categorical Factors	*p*-value
Urgency of Operation	0.1199
Re-operation	0.8097
Severe Chronic Lung Disease	0.4225
Dialysis	0.1915
Diabetes	0.5321
Age	0.4274
Ejection fraction	0.6072

## Discussion

High risk patients with concomitant aortic stenosis and mitral regurgitation are left with uncorrected mitral valve disease after their TAVR. As the presence of significant mitral regurgitation is associated with reduced survival for patients undergoing TAVR, it is unclear if such patients would be better served with surgical correction of both the aortic and mitral valves rather than TAVR alone. The purpose of this study was to determine the outcomes of high risk patients who would have been candidates for TAVR who underwent surgical aortic valve replacement and mitral valve surgery.

Prior studies have shown increased postoperative mortality and morbidity with concomitant aortic and mitral valve surgery [12–16]. Maleska et al. reports a 91% and 71% survival rates at 30 days and 1 year, respectively, for octogenarians undergoing simultaneous aortic and mitral valve replacement [17]. However, the mortality risk of double valve surgery in patients with concomitant aortic and mitral valve disease who may be considered for TAVR remains unclear. This study is the first to look at outcomes of high risk patients with severe aortic stenosis undergoing surgical aortic valve replacement and mitral valve surgery. Although the actual operative mortality rate of 11.6% in our study cohort was lower than the total predicated risk of mortality rate for AVR alone of 14.6%, the long term 1 year and 2 year survival rate was only 67% and 58%, respectively. Therefore, our study demonstrates that, despite reasonable operative survival rates, high risk patients with concomitant severe aortic stenosis and mitral valve disease have poor long term prognosis even after surgical correction of both valvular abnormalities.

Numerous studies have demonstrated increased mortality in patients with significant MR undergoing TAVR [6, 8, 18, 19]. A meta-analysis of eight studies including 8015 patients found increased overall 30-day and 1-year mortality with an odds ratio of 1.49 and 1.92, respectively, in TAVR patients with significant MR as compared to those without [4]. Another meta-analysis of 16 studies and 13,672 patients demonstrated a similar increase in early and overall all-cause mortality in patients with significant MR as compared to patients without MR or with non-significant MR with an odds ratio of 2.17 and a hazard ratio of 1.81, respectively [5]. A large multicenter study of 1110 patients by Cortes et al. found that patients with significant pre-TAVR MR had a 3 fold increase in 6 month mortality as compared with patients without significant pre-TAVR MR (35.0% vs. 10.2%, *p* < 0.001) [2]. The same study found that although the degree of MR have been shown to improve in up to 60% of patients undergoing TAVR, improvement of baseline MR was not associated with improved cardiac mortality [2]. Mavromatis et al. reported a one year motality and heart failure rehospitalization rate of 28.0% and 23.4%, respectively, in patients who underwent TAVR with severe MR [9]. A study by Toggweiler et al. looked at the outcomes of patients undergoing TAVR with concomitant severe MR [6]. With 49% having previous open-heart surgery, 26% having chronic obstructive pulmonary disease, 35% having pulmonary hypertension, and an average STS risk score of 9.7%, the study population in their study was comparable to that of the current study. They report a survival of 84%, 65% and 59% at 30 days, 1 year and 2 years, respectively, which is comparable to the survival rate of 77%, 67% and 58% reported in our study [6].

Although significant MR is associated with increased short and long term mortality in patients undergoing TAVR, they are comparable to the mortality rates for our study cohort of similar patients undergoing concomitant aortic and mitral valve surgery. As such, surgical correction of both valves may not alter the long term outcomes in these high risk patients as compared to TAVR alone. Furthermore, the advent of percutaneous interventions for treating mitral valve disease may offer appropriate patients percutaneous alternatives for the management of residual MR after TAVR [20]. Percutanous edge to edge mitral valve repair with MitraClip (Abbott Vascular, Menlo Park, CA) has been the most widely used and studied for the treatment of significant MR after TAVR. Rudolph et al. reported a case series of 11 patients receiving both TAVR and MitraClip therapy with successful reduction of MR severity to <2+ in 10 patients [21]. Another case series of 12 patients who underwent MitraClip placement after TAVR by Kische et al. reported a 100% procedural success rate with MitraClip with no patients with greater than 1+ MR after MitraClip. All patients in that series also experienced functional improvement after MitraClip [22]. A contemporary series of 14 patients undergoing MitraClip after TAVR reported a 92.9% procedural success rate with 21.4% of patients with recurrent 3+ MR at one year [23]. Interestingly, the reported one year survival rate of 66.5% in that series is similar to that of our study [23]. This may further support that correction of the mitral regurgitation may not offer survival benefit in this select high risk patient population.

There are several limitations to this study. First, as an STS score is unable to be calculated for double valve surgery, we used the STS score for isolated AVR as the basis for expected mortality risk for risk stratification. Although this inevitably underestimates the surgical risk for double valve surgery, its use is justified for the purpose of this study as the same score would be used for the risk stratification of patients with concomitant mitral and aortic valve disease being considered for TAVR. Secondly, as this study was limited to patients to high risk patients undergoing concomitant surgical aortic valve replacement and mitral valve surgery, the results of this study may not be applicable to intermediate and low risk patients undergoing similar surgeries. Lastly, as with all single center studies, the results of this study may not be generalizable to all institutions.

## Conclusion

In conclusion, high-risk patients with severe aortic stenosis and mitral valve disease undergoing concomitant surgical aortic valve replacement and mitral valve surgery have similar long term survival as that described for such patients undergoing TAVR. Surgical correction of double valvular disease in this patient population may not confer mortality benefit compared to TAVR alone.

## Abbreviations

LOS: length of stay
MR: mitral regurgitation
STS PROM: Society of Thoracic Surgery predicted risk of mortality
TAVR: transcatheter aortic valve replacement
